# New method to improve the quality of vision in cataractous keratoconus eyes

**DOI:** 10.1038/s41598-020-76977-4

**Published:** 2020-11-18

**Authors:** Juan J. Miret, Vicente J. Camps, Celia García, María T. Caballero, Dolores de Fez, David P. Piñero

**Affiliations:** 1 Grupo de Óptica y Percepción Visual (GOPV), Department of Optics, Pharmacology and Anatomy, University of Alicante, Crta San Vicente del Raspeig s/n, San Vicente del Raspeig, 03690 Alicante, Spain; 2 Department of Ophthalmology, Vithas Medimar International Hospital, Alicante, Spain

**Keywords:** Biophysics, Diseases, Medical research, Optics and photonics

## Abstract

To analyze using optical simulations if the proper use of a segmented intraocular lens (IOL) can improve the visual outcomes compared to the implantation of a spherical monofocal IOL. The wavefront profile of the Mplus (Oculentis) and a monofocal IOLs with the phase transformation introduced by each IOL were calculated using a Hartmann-Shack wavefront sensor. In addition, the wavefront profile of schematic eye models of various keratoconus conditions was obtained and was propagated to the IOLs. The optical performance of such combination was obtained after combining ray tracing and Fourier optics. A pre-clinical validation was also evaluated incorporating clinical data from three different keratoconus eyes of three patients. The implantation of the Mplus IOL can compensate or reduce the overall coma of the eye with keratoconus improving the quality of vision compared with a spherical monofocal IOL due to lower displacements of the retinal image or tilting in keratoconus. All theoretical simulations were confirmed afterwards by mean of a preclinical validation. The use of a standard toric segmented IOL with a proper orientation and selection of the addition can improve the optical quality of the keratoconus eye compared to the use of a monofocal spherical IOL.

## Introduction

**Figure 1:**
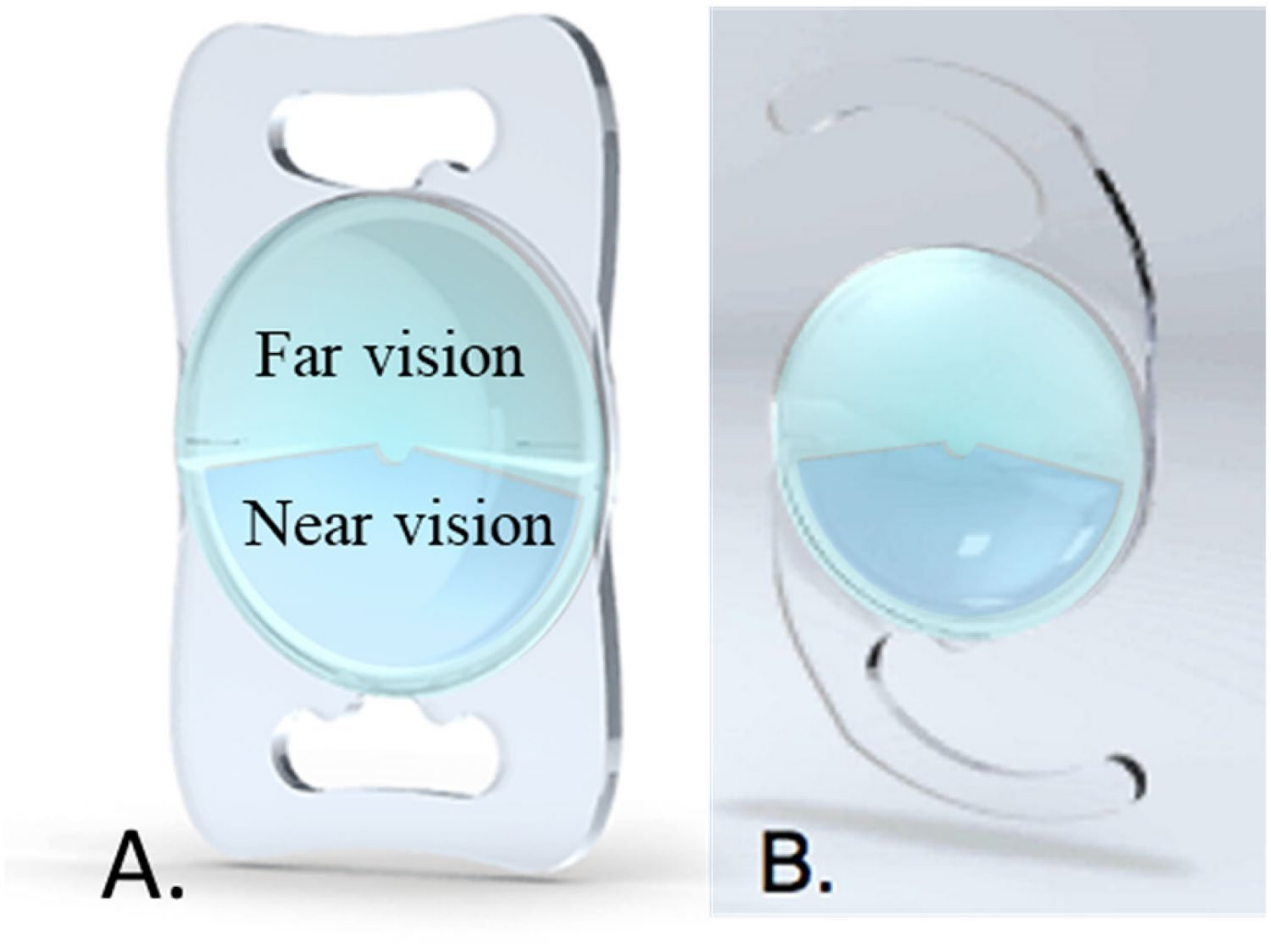
Distance and near sectors of a aspherical nonrotational symmetric multifocal IOL. (**A**) Plate-haptic model. (**B**) Model with C-loop haptic.

Rotationally asymmetric refractive multifocal intraocular lenses (IOLs) have been used in modern lens-based surgery for the past 10 years. These IOLs provide two foci due to their bifocal refractive design. This design incorporates a segment for near vision and the rest of the optics is used for far vision. Therefore, the IOL has two sections, creating only 1 transition zone (see Fig. 1)^1^. This asymmetrical design could introduce a new possibility in the implantation depending on the position of the near segment. With this type of IOLs, the recommended placement of the segment for near is inferiorly with slight nasal deviation; however, some studies propose other orientations that could also be valid in terms of visual quality^2–4^. Wit et al.^4^ found that a superotemporal placement of the IOL was well tolerated, concluding that the positioning of the near add did not significantly affect objective or subjective visual function parameters. McNeely et al.^2^ obtained high overall quality of vision using a combination of superotemporal placement of the near segment in the dominant eye with inferonasal placement of the near segment in the fellow eye. In a more recent study, Liu et al.^3^ found different optical performance of rotationally asymmetric refractive IOLs depending on the aperture and the segment decentration. These authors found that the optical quality was better for the aperture of 4.5 mm compared to 3.0 mm for a centered asymmetric IOL, at near focus for a near–horizontal decentration and at far focus for a distance–horizontal decentration. Montes-Mico et al.^5^, using an in vitro experiment, shown that tilt and decentration had a significant impact on optical quality for both symmetric and asymmetric multifocal IOLs, being more severe such impact for the asymmetric IOLs.

Keratoconus is a condition in which the cornea assumes a conical shape because of a progressive corneal thinning. This corneal change induces irregular astigmatism with or without myopia and an increment of high order aberrations. An impairment of both quantity and quality of vision is produced^6^. It is known that this corneal disease can lead to very significant wavefront errors, with specifically an important increase of coma-like aberrations^7^. Maeda et al.^8^ found several years ago a prevalence of coma-like aberrations over spherical-like aberrations in keratoconic eyes, mainly due to the displacement of the apex. In fact, coma-like aberrations were found to be 2.32 times larger than spherical-like aberrations in keratoconic eyes (RMS coma-like = 1.88 ± 1.16 µm vs. RMS spherical-like = 0.70 ± 0.55 µm).

Some attempts to reduce aberrations in keratoconus eyes have been conducted. Suzaki et al., using a standardized asymmetric vertical power distribution soft contact lens significantly reduced HOAs and mainly the coma^9^. In a more recent study Nilagiri et al. also indicated that optical performance of keratoconic eyes are significantly better with Rigid Gas Permeable Contact Lenses than with spectacles because the HOAs are lower^6^. The question is if these type studies could be addressed in intraocular lenses.

Some studies have shown that segmented rotationally asymmetric multifocal IOLs induce significant amounts of intraocular primary coma which is usually attributed to its vertical asymmetric optical geometry^10,11^.

Recently, our research group measured the aberrations of the Lentis Mplus X LS-313 MF30 IOL using a Hartmann-Shack wavefront sensor implemented in an optical bench. In this study, we found that the Mplus IOL provided vertical coma if the segment was placed inferiorly. A value of − 0.22 µm of vertical coma was obtained for a 3-mm pupil size and a value of − 0.40 µm for a 4-mm pupil size^12^. In cataractous keratoconic eyes, the usual procedure is to implant spherical monofocal IOLs, not compensating for the higher order aberrations (especially coma) and compromising then the possibilities of improvement of the quality of vision. In 2008, Chen et al.^13,14^ found that the coma aberration of the first and second corneal surfaces were of opposite sign, compensating the coma component of the second surface partially the negative coma of the first surface. Considering that negative coma is the dominant high order aberration in keratoconus eyes and that this coma could be compensated generating positive coma, our aim was to analyze using optical simulations if the proper use of a standard segmented IOL can diminish the level of primary coma in keratoconus, promoting an improvement of the visual outcomes compared to the implantation of a spherical monofocal IOL. For such purpose, data and procedures previously reported in two already published papers were used. First, the novel method used to simulate the optical performance of presbyopia-correcting IOLs in a previous study of our research group was adapted for the simulations of the current study^15^. Second, data from different schematic keratoconic eye models described by Tan et al.^13^ were used.

As far as we known, only one study has analysed the possibility of implanting segmented IOLs in keratoconus. Ouchi et al.^16^ evaluated the postoperative outcomes of complicated cataract eyes with coexisting ocular pathologies that underwent cataract surgery with implantation of a refractive multifocal intraocular lens (MIOL) containing a surface embedded near section. Two keratoconic eyes were included in this series and the authors concluded that the implantation of this type of IOL was effective in such cases. In another hand, in a computational study Schröder et al. proposed the use of personalized freeform IOLs in order to reduce the aberrations in cataractous keratoconic eyes. They stablished that this option may become a promising option for the correction of advanced aberrations in eyes with non-progressive keratoconic corneal tomography pattern^17^.

## Methods

As commented above, the methodology used in this study was based on a previous study in which the optical performance of three presbyopia-correcting IOLs (Mplus IOL included) implanted in eyes with previous laser refractive surgery was simulated^15^. In this study, the wavefront profile of the rotationally asymmetric refractive IOL Mplus (Oculentis) and the phase transformation introduced by this design were calculated using a Hartmann-Shack wavefront sensor following the guidelines established by the ISO11979-9^18,19^.

Tan et al.^13^ proposed schematic eye models of various keratoconus conditions fitting their surfaces to two-dimensional Gaussian profiles. The keratoconus cornea model was simulated using 5 cone parameters, (x_o_, y_o_, σ_x_, σy, h_o_) where h_o_ represents the peak height of the cone, (x_o_, y_o_) the cone’s center location with respect to the visual axis and (σ_x_, σy) the corresponding dimensions where the height drops to e^−1/2^ of the cone’s peak height. Based on the statistical distribution of measured cone volumes and the shape-correlated eccentricity, they proposed 14 cones classified in four degrees (mild, moderate, advanced and severe). The authors based on their studies considered that the average cone tend to be located inferiorly and temporally to the visual axis (that is y_o_  = − 0.9 ± 0.5 mm and x_o_ = 0.4 ± 0.7 mm).

Following our methodology, the corneal wavefront profile of each simulated keratoconus was obtained and was propagated to the IOL which was introduced as a phase element. The optical performance of such combination was obtained after combining ray tracing and Fourier optics (Zemax, LLC Washington, USA and MATLAB, The MathWorks, Natick, MA).

As commented above, the Lentis Mplus was found to produces negative vertical coma when the near vision segment was located inferiorly^12^. Therefore, if the segment would be located superiorly, the coma would be expected to change its sign should compensate the type of coma normally associated to keratoconus. Considering the average cone location, the segment of the Mplus was located superiorly and oriented in order to reduce or compensate the coma produced by keratoconus. Specifically, the Mplus symmetry axis (Y-axis) is rotated at an angle given by $$ \theta = tan^{-1} (\frac{keratoconus primary horizontal coma}{keratoconus primary vertical coma})$$, where *θ* is positive for clockwise rotation. Furthermore, in this way, the tilt introduced by keratoconus is also reduced and therefore the displacement of the final image.

**Figure 2:**
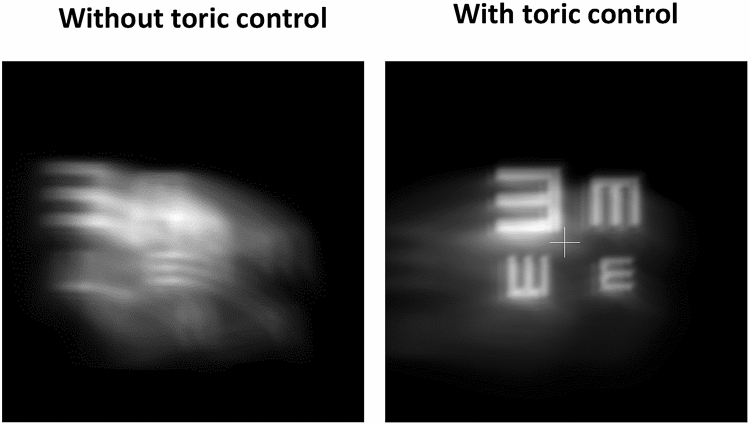
Simulation of the retinal image obtained with and without control of regular astigmatism.

Although coma is the principal high order aberration induced by keratoconus, elevated regular corneal astigmatism is also induced. To deal with it, toric IOLs are normally used. As the aberrometric profile of the non-toric model of the Mplus IOL had been previously characterized, a toric ophthalmic lens was added to our simulation approach to control the potential impact of keratoconus regular corneal astigmatism. As seen in Fig. 2, the control of this astigmatism produces a clear improvement of the image quality.

**Figure 3:**
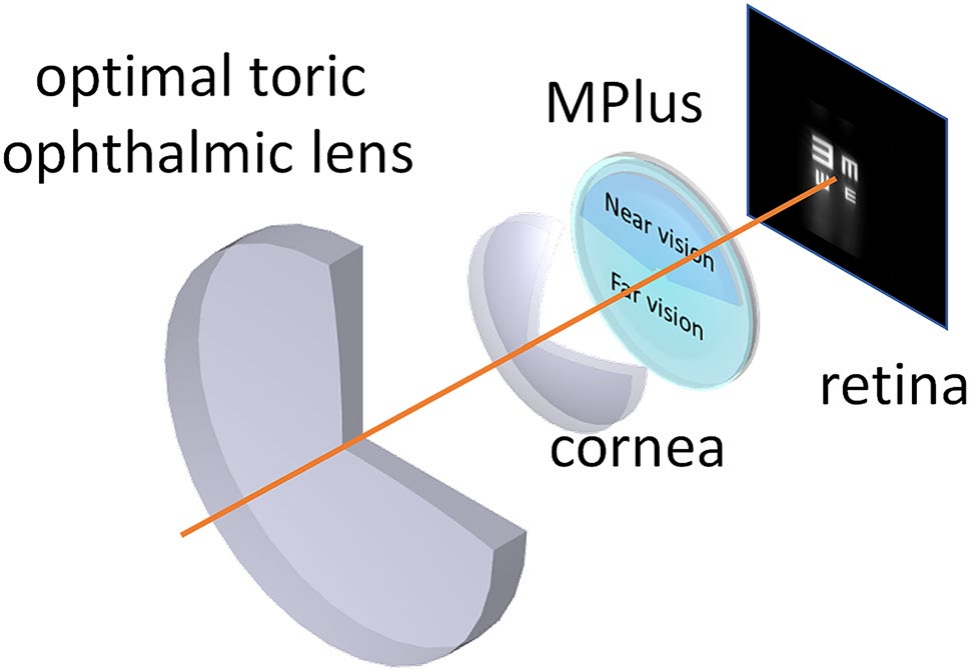
Scheme of the simulation system used in the current study.

In summary, the simulation system consisted of a toric ophthalmic lens, the keratoconic cornea, the IOL and the retinal plane (see Fig. 3). As commented above, the standard solution for cataractous keratonic eyes is to implant a monofocal IOL. In order to assess the potential benefits of using a rotationally asymmetric refractive IOL, the simulations were also performed for a standard spherical monofocal IOL. Specifically, the results with the Akreos Adapt-AO IOL (Bausch & Lomb) of 20 D and with the Lentis Mplus X LS-313 MF30 IOL (Oculentis) of 20 D and 3 D of addition were compared.

Once performed the simulations considering the keratoconus eye models, the effect of the implantation of the rotationally asymmetric refractive IOL was also evaluated incorporating clinical data from three different keratoconus eyes of three patients revised at the Department of Ophthalmology of the Vithas Medimar International Hospital (Alicante, Spain).

## Results

**Figure 4:**
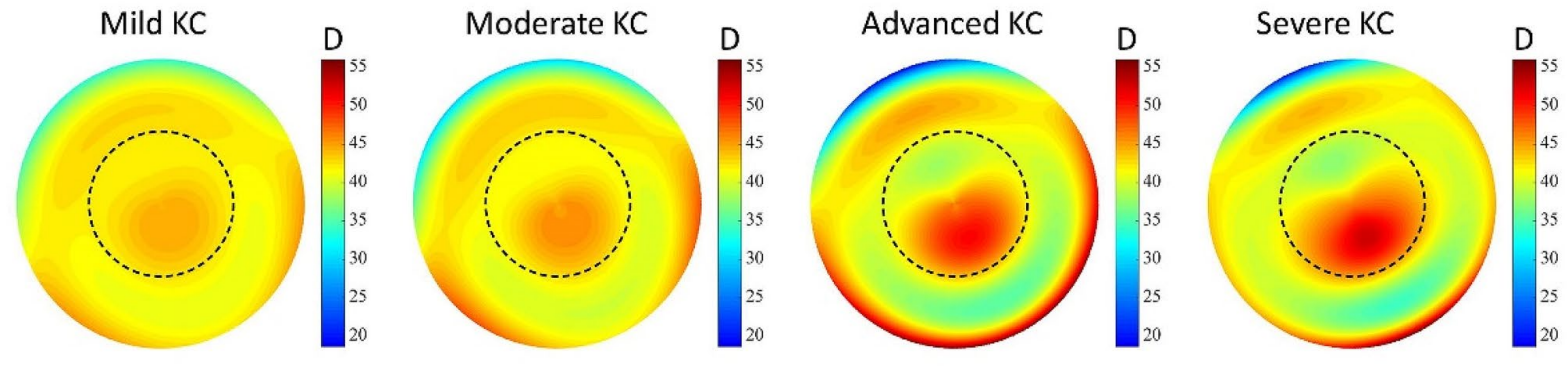
Tangential curvature maps of the selected keratoconus eye models according to Tan et al.

Out of the 14 synthetic keratoconus (KC) eye models proposed by Tan et al.^13^, we have chosen one mild (number 2), one moderate (number 5), one advanced (number 10) and one severe KC (number 12) cases were considered. In Fig. 4, the simulated topographies of these KC cases are shown.

**Figure 5:**
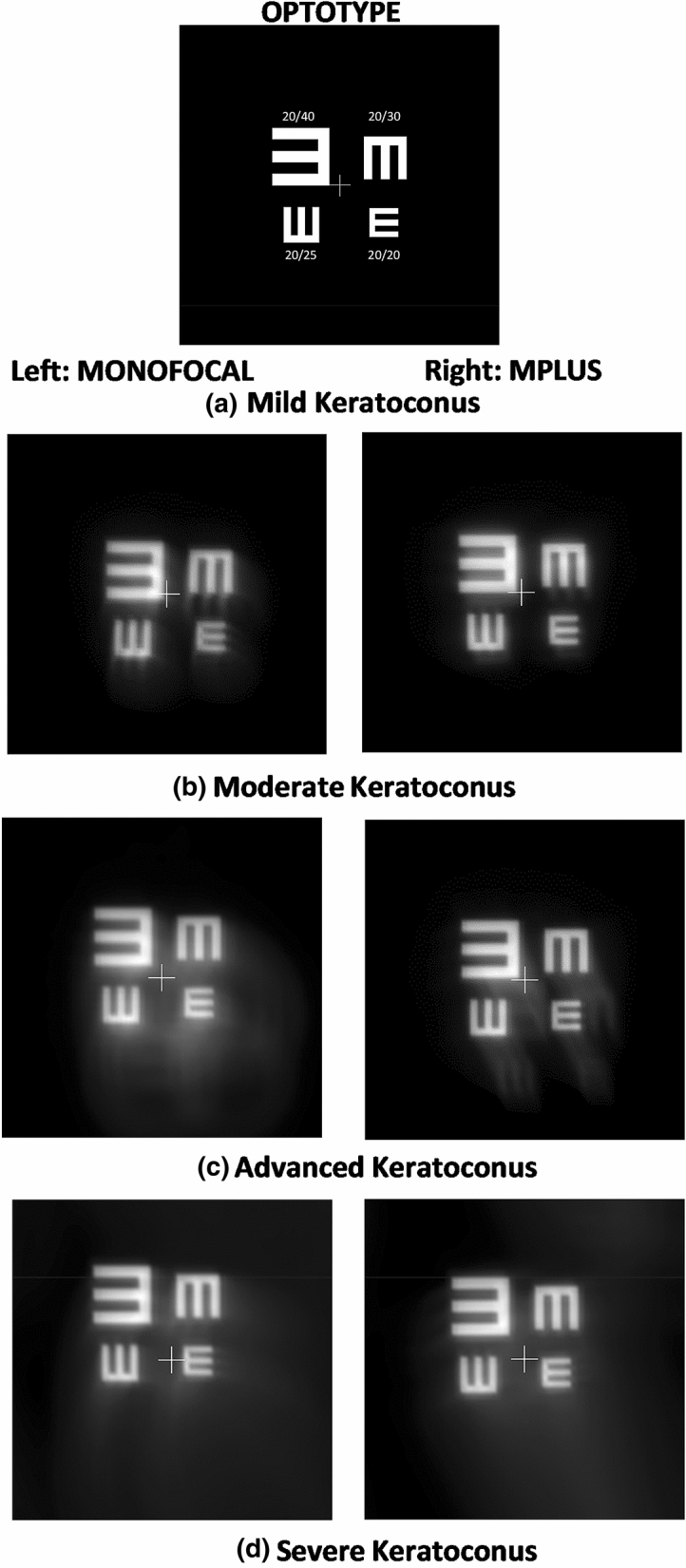
Optotype simulations for the selected keratoconus eye models according to Tan et al. implanted with a monofocal or Mplus IOL.

Figure 5 shows the simulation of the perceived optotypes when a monofocal or Mplus IOLs were implanted. For better understanding of differences, optotypes from 20/40 to 20/20 visual acuity were represented. Furthermore, a cross was drawn in the center of the optotype in order to analyze the displacement of the image of the optotypes. As seen in Fig. 5, the use of the Mplus IOL always provided better results compared to the monofocal spherical IOL. With the monofocal IOL, the effect of coma was clearly observed for all KCs type, inducing an asymmetric distortion of the image. However, if the Mplus IOL was implanted, the coma aberration was compensated for the mild KC case and reduced for the rest of KCs. This comatic reduction decreased as the KC grade increased. This behavior can be explained considered the data summarized in Table 1. The vertical coma generated by the Mplus IOL was + 0.169 µm and the horizontal − 0.074 µm for a 6-mm pupil, and they compensated the vertical and horizontal coma components that were present in the mild KC case (− 0.170 and + 0.074 µm respectively). However, this amount of coma produced by the Mplus IOL was not enough to compensate the entire coma aberration that was present in the rest of KC cases evaluated.

**Table 1:** Primary coma data associated to the Mplus IOL and to the KC cases considered for the simulations.

Microns (µm)	Mplus	Mild KC	Moderate Kc	Advanced KC	Severe KC
y-coma	0.169	− 0.170	− 0.314	− 0.888	− 1.016
x-coma	− 0.074	0.074	0.140	0.396	0.452

Another important effect of the IOL was the displacement of the optotype image regarding the middle point of the test (represented by a cross). As seen in Fig. 5, the Mplus IOL provided a lower displacement of the image compared to the spherical monofocal IOL. This displacement increased as the severity of the KC increased.

### Pre-clinical validation

The methodology developed for the previous simulations was validated by using it in real cases considering the real corneal topography data instead of modelled data. The wavefront profile from the real corneal topography was obtained in 3 eyes of 3 different patients:

- Case 1: right eye, male 27 years old, manifest refraction + 0.75 sph − 2.00 × 90º cyl, corrected distance visual acuity 0.05 logMAR, mean anterior keratometry 43.63 D, minimum corneal thickness 517 μm.
- Case 2: right eye, female 34 years old, manifest refraction − 7.00 sph − 1.50 × 40º cyl, corrected distance visual acuity 0.00 logMAR, mean anterior keratometry 46.12 D, minimum corneal thickness 483 μm.
- Case 3: left eye, female 35 years old, manifest refraction − 9.50 sph − 3.00 × 145º cyl, corrected distance visual acuity 0.5 logMAR, mean anterior keratometry 53.13 D, minimum corneal thickness 401 μm.

This study was conducted following the tenets of the Declaration of Helsinki and all patients signed a consent approved by the Ethics Committee of the University of Alicante allowing the use of their data for this retrospective analysis.

**Figure 6:**
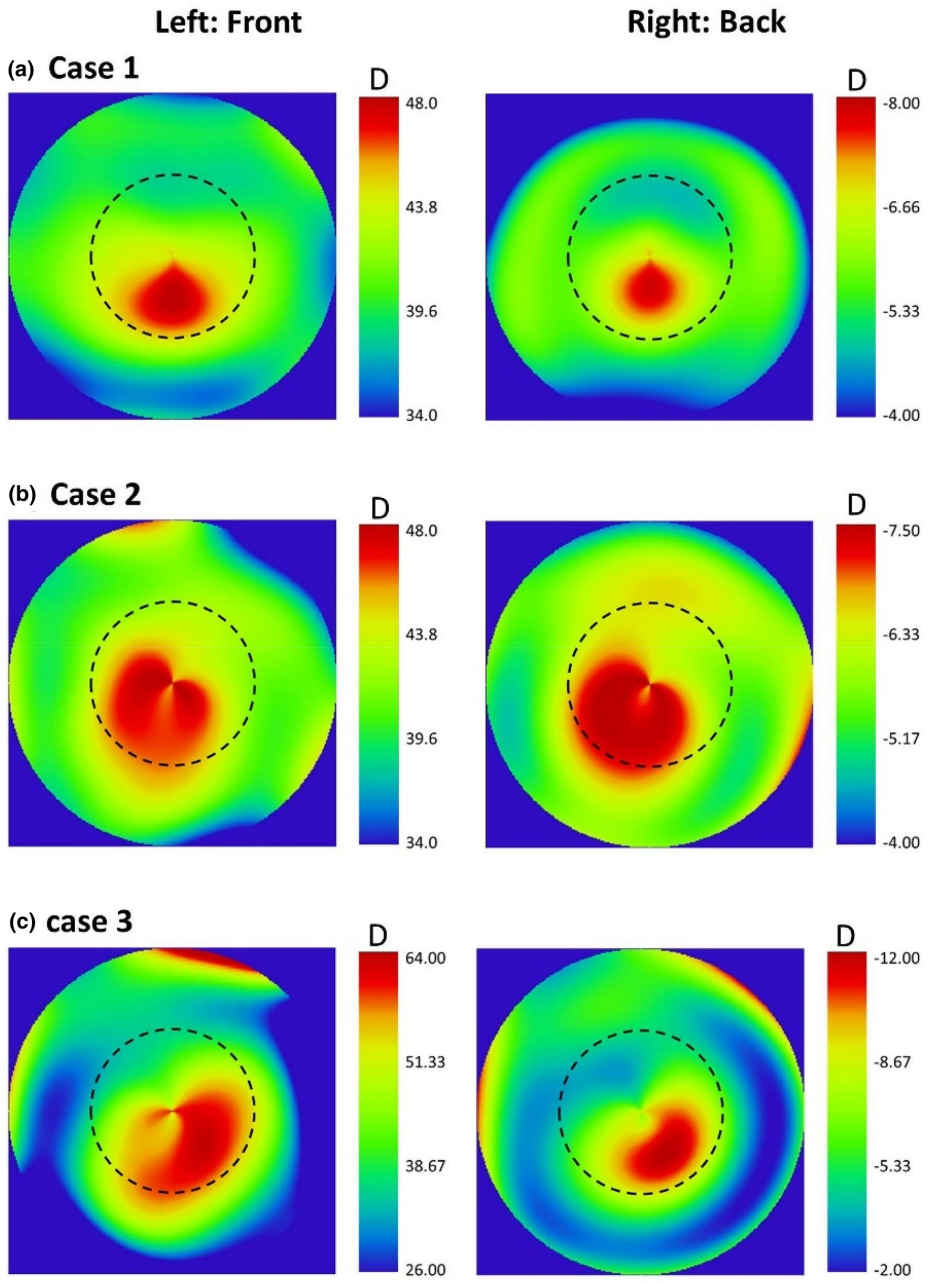
Tangential curvature maps in diopters of the 3 clinical cases evaluated (**a**–**c**) Left: front corneal surface; right: posterior corneal surface.

The corneal wavefront profile of each of these cases was propagated through the IOL using the ray tracing software. In these calculations, both front and back corneal topographies were considered. Figure 6 shows the tangential curvature maps of anterior and posterior corneal surfaces of these three KC patients of different grades. According to the Amsler-Krumeich grading system, case 1 and 2 corresponded to a keratoconus grade 1 and case 3 to a keratoconus grade 3.

**Figure 7:**
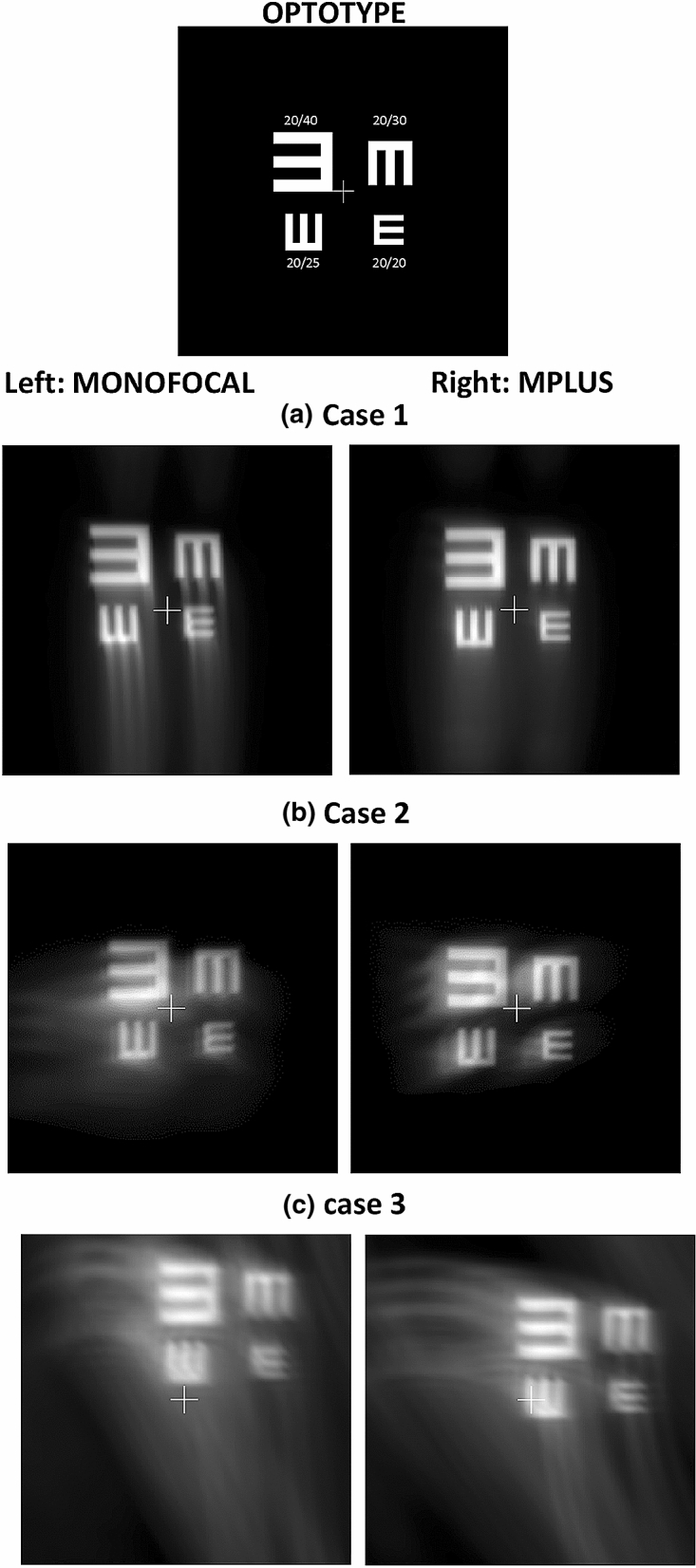
Optotype simulations for the three keratoconus clinical cases evaluated with a monofocal or Mplus IOL.

Figure 7 confirmed the same trend observed using simulated KC eye models. The Mplus IOL always provided better results than the monofocal IOL, with a potentially improved visual acuity and less displacement of the retinal image of the optotype. The degradation of vision was more relevant, even with the Mplus IOL implanted, as the severity grade of keratoconus increased.

## Discussion

The simulations performed in the current study have shown that a proper rotation of the vertical axis of a standard segmented IOL as the Mplus IOL by aligning it with the highest power value of the cone peak induce levels of primary coma with an opposite sign than that corresponding to the KC. Therefore, the implantation of the Mplus IOL can compensate or reduce the overall coma of the eye with keratoconus improving the quality of vision in comparison with the implantation of a spherical monofocal IOL. Likewise, the implantation of this multifocal IOL is associated to lower displacements of the retinal image or tilting in keratoconus. This image displacement or tilting could produce a clinically relevant reduction of the visual field as well as an alteration of the patient’s visual perception, especially in the most advanced keratoconus cases. According to our simulations, the location of the IOL near segment required to compensate the level of coma associated to keratoconus would be superior, confirming that the conventional inferior location of the segment would worsen the quality of vision compared to a monofocal IOL in keratoconus. If the vertical axis does not exactly match the cone peak, the visual results expected would be worse according to our simulations, with an optical quality among those obtained with a monofocal IOL and the Mplus with the most optimized orientation of the near segment. Our results somehow corroborates the study presented by Ouchi et al.^16^ that concluded that the implantation of a segmented IOL was effective for treating two cataractous keratoconic eyes.

All theoretical simulations were confirmed afterwards by mean of a preclinical validation in 3 real keratoconus cases. As seen in Fig. 7, the implantation of the MPlus IOL provided a good outcome in terms of optical quality for cases 1 and 2, obtaining simulations compatible with the achievement of a visual acuity of 20/20, with negligible loss of visual field. In these two cases, the position of the cone peak is inferior close to the 90º meridian, with primary coma aberration that could be easily compensated with a segmented IOL with the near segment positioned upwards. However, in the case 3 (severe KC), differences in terms of optical quality achieved between Mplus and monofocal IOL were less evident due to the asymmetrical position of the cone peak, among other factors. In any case, image tilting was less relevant with the Mplus IOL, with the potential of affecting less to the visual field.

Another relevant point analysed in the current simulation study was to consider the second corneal surface to estimate the real contribution of the primary coma aberration of the cornea in order to understand the real level of aberration that had to be compensated by the segmented IOL. As shown in Fig. 6, all keratoconus clinical cases showed a similar distribution of optical power in both surfaces but with opposite sign, generating then coma aberration of opposite sign. Therefore, some level of compensation of coma aberration is present between anterior and posterior corneal surfaces which is consistent with the correlation in shape between surfaces in healthy and keratoconus eyes^20,21^. This means that to consider only the first corneal surface to optimize intraocular implants in keratoconus would lead to an overcorrection of coma aberration. This conclusion is in line with those extracted by Chen et al.^14^ about the compensation of coma between the first and second corneal surface in the KCs.

In the current simulation study, the Mplus IOL with an addition of + 3 D was considered. Possibly, the modification of the addition may allow for the compensation of different amounts of primary and secondary coma, providing more possibilities of optimization. Indeed, a proper combination of addition and axis rotation for each patient would require if this type of IOLs are considered as a potential treatment option in keratoconus eyes undergoing cataract surgery. Our research in a way follows the idea proposed by Schröder et al.^17^ because we have confirmed that keratoconic eyes could benefit more from aberration correction with customized intraocular lenses (IOLs) than normal cataractous eyes. As these authors showed using personalized numerical ray tracing models that characterize corneal surfaces by Zernike coefficients and optimize the IOL, it is possible to reduce the final aberrations of the eye. The difference lies that we proposed the use of a segmented IOL that already exists. According to our simulations, this optimization of the optical quality in mild to moderate keratoconus is viable, being more difficult this type of optimization in severe cases. Experimental studies are needed to confirm if the results of these preliminary simulation evidences can be clinically feasible.

Finally, it should be considered that the regular component of astigmatism was compensated in our simulation with an optimized toric ophthalmic lens for each case. This compensation is crucial to obtain good visual results with a segmented IOL in order to optimize the quality of vision in cataractous keratoconic eyes undergoing cataract surgery. Therefore, the segmented design of the IOL must be combined with a toric surface for a completely optimized visual rehabilitation. As advantage, this solution requires the use of standard toric segmented IOLs in front of the proposal of using custom IOLs^17^ or the adaptation to contact lenses^6,9^.

In conclusion, a new possibility to improve the ocular optical quality in keratoconus eyes is presented. The implantation of an optimized toric segmented IOL with a proper orientation and selection of the addition can improve the optical quality of the keratoconus eye compared to the use of a monofocal spherical IOL. Further clinical validation is required in order to confirm these results.
